# Data Reduction in Phase-Sensitive OTDR with Ultra-Low Sampling Resolution and Undersampling Techniques

**DOI:** 10.3390/s22176386

**Published:** 2022-08-24

**Authors:** Feihong Yu, Liyang Shao, Shuaiqi Liu, Weijie Xu, Dongrui Xiao, Huanhuan Liu, Perry Ping Shum

**Affiliations:** 1 Department of Electrical and Electronic Engineering, Southern University of Science and Technology, Shenzhen 518055, China; 2 Peng Cheng Laboratory, Shenzhen 518005, China; 3 State Key Laboratory of Analog and Mixed-Signal VLSI, University of Macau, Macau 999078, China

**Keywords:** phase-sensitive optical time-domain reflectometry, distributed acoustic sensing, data reduction, ultra-low sampling resolution, undersampling

## Abstract

Data storage is a problem that cannot be ignored in the long-term monitoring of a phase-sensitive optical time-domain reflectometry (Φ-OTDR) system. In this paper, we proposed a data-reduction approach for heterodyne Φ-OTDR using an ultra-low sampling resolution and undersampling techniques. The operation principles were demonstrated and experiments with different sensing configurations were carried out to verify the proposed method. The results showed that the vibration signal could be accurately reconstructed from the undersampled 1-bit data. A space saving ratio of 98.75% was achieved by converting 128 MB of data (corresponding to 268.44 ms of sensing time) to 1.6 MB. The proposed method led to a potentially new data-reduction approach for heterodyne Φ-OTDR, which also provided economical guidance for the selection of the data-acquisition device.

## 1. Introduction

Phase-sensitive optical time-domain reflectometry (Φ-OTDR)-based distributed acoustic sensing technology has been extensively researched in recent years, and it has already been deployed in many practical applications [1,2], such as geological monitoring [3,4], perimeter security [5,6], traffic sensing [7], and so on. In a Φ-OTDR system, a probe pulse is launched into the sensing fiber and simultaneously stimulates the Rayleigh backscattering (RBS) light. The physical parameters of the RBS light would be affected if there were external vibrations acting on the sensing fiber. After collecting the RBS traces, the locations of the vibrations along the fiber can be deduced by utilizing proper demodulation methods [8,9]. Compared to the conventional direct detection structure that can only locate disturbances, a heterodyne detection scheme has a higher signal-to-noise ratio (SNR) and the capability of demodulating the vibration waveform, which enables further analysis of the vibrations [10,11,12].

Generally, despite the probe pulse, there is a continuous local reference light that beats with the RBS light in heterodyne Φ-OTDR, resulting in a bandpass signal. The center frequency of the beat signal is dependent on the frequency shift induced by the modulator, and its typical value is in the range of tens of megahertz to one or two hundred megahertz [13,14,15,16,17]. According to the Nyquist–Shannon sampling theorem, the sampling rate of the data-acquisition device should be at least twice the highest frequency of the beat signal so as to avoid the aliasing of the captured signal. For example, if the upper cutoff frequency is 80 MHz, then the sampling rate of the data-acquisition device should be at least 160 MSa/s. To ensure the quality of the captured beat signal, the sampling rate can even be increased to three times its maximum frequency, i.e., 240 MSa/s, or higher. In [15,18], the center frequencies of the beat signal were 80 MHz and 200 MHz, and the corresponding applied sampling rates were 500 MSa/s and 1 GSa/s, respectively. To achieve a sampling rate of several hundred megahertz, a high-performance data-acquisition device was essential, which raised the cost of the system.

On the other hand, the sampling resolution is another critical parameter for a data-acquisition device. The data-acquisition step is an analog-to-digital conversion process, and the sampling resolution is the parameter used to depict the precision of the captured samples. The number of bits is generally used to qualitatively delineate the resolution of the sample, and the more bits used to represent a sample, the closer that sample is to the value of the real analog signal. Basically, the sampling resolution in a Φ-OTDR system is above 8 bits, and some systems employ a higher sampling resolution [19,20,21]. Although a high sampling resolution allows the captured beat signal to more accurately resemble the real signal, the high data volume it requires is an issue that has to be taken into consideration. Supposing a data-acquisition device has a 250 MSa/s sampling rate and a 16-bit resolution, then it will produce approximately 476.84 MB of data per second, making it almost impossible for it to work continuously due to the limitations of the write speed and capacity of the storage media.

Currently there are two proven approaches for solving the data-storage problem in the Φ-OTDR system [22,23]. One approach is to first demodulate the captured beat signal and then extract the specific phase results on the distance axis according to the spatial resolution of the system [22]. This method stores the phase demodulation results after extraction, which can significantly reduce the data volume; however, this method sacrifices the dense sampling points along the distance axis and also deteriorates the accuracy of the perturbation localization. Another approach is to average the collected RBS traces along the time axis, which can increase the trace SNR while reducing the number of traces that need to be stored [23]. This method is very effective in low-frequency detection applications; however, after averaging the RBS traces, the frequency response range of the Φ-OTDR system also decreases, thus this method is not suitable for other application scenarios.

As mentioned above, the beat signal is a bandpass signal, and its center frequency is given by the frequency shift of the modulator. Based on this feature, researchers have applied the undersampling theory to heterodyne Φ-OTDR systems and have successfully captured a beat signal with a 200 MHz center frequency using a sampling rate of 71 MSa/s [24]. In addition, our previous work proved that an ultra-low sampling resolution (e.g., 1 bit) could be employed in heterodyne Φ-OTDR systems [25]. In this paper, we proposed a data-reduction solution for heterodyne Φ-OTDR using an ultra-low sampling resolution and undersampling techniques. Storing the undersampled low-resolution data could significantly minimize the data volume of a system, facilitate data transmission, and also reduce the cost of the data-acquisition device as well as storage media. This paper is presented as follows. Firstly, the center frequency and bandwidth of the RBS signal were deduced, then we describe the data-reduction mechanism. In addition, the operation principles of an ultra-low sampling resolution and undersampling techniques are given. Finally, experiments were carried out to verify the feasibility of the proposed solution.

## 2. Operation Principles

### 2.1. Conventional Heterodyne Φ-OTDR

For conventional heterodyne Φ-OTDR, the backscatter impulse model can be employed to describe the RBS signal [26,27]. Supposing the pulse width of the injected probe pulse is $T_{p}$, the electric field of the RBS signal can be written as follows:

(1) $$\begin{array}{c} E_{RBS}\left(t\right)=\sum_{i=1}^{N}E_{0}a_{i}\exp\left(-\alpha \nu \tau_{i}\right)\exp\left\{j\left[2\pi \left(f_{0}+\Delta f\right)\left(t-\tau_{i}\right)+\varphi_{0}\right]\right\}\cdot \\ \;\;\exp\left[j\varphi \left(\tau_{i}\right)\right]\mathrm{rect}\left[\left(t-\tau_{i}\right)/T_{p}\right] \end{array}$$

where rect() is the rectangular function; $a_{i}$, $\tau_{i}$, and $\varphi \left(\tau_{i}\right)$ are the amplitude, relative delay, and phase of the *i*-th scatter respectively, and N is the total number of scatterers; $E_{0}$, $f_{0}$, and $\varphi_{0}$ represent the amplitude, frequency, and initial phase of the incident light respectively; α refers to the optical power attenuation coefficient of the sensing fiber; ν denotes the velocity of light in the fiber; Δf is the frequency shift of the modulator. In addition, the electric field of the local oscillator (*LO*), i.e., the local reference light, can be written as:

(2) $$E_{REF}=E_{LO}\exp\left[j\left(2\pi f_{0}t+\varphi_{0}\right)\right]$$

where $E_{LO}$ is the amplitude of the reference light. The beat signal is the result of mixing the RBS light and the reference light, and the intensity of the detected beat signal can be written as follows:

(3) $$\begin{array}{cc} I\left(t\right)\propto & \sum_{i=1}^{N}E_{0}E_{LO}a_{i}\exp\left(-\alpha \nu \tau_{i}\right)\cdot \\ & \cos\left[2\pi \Delta ft-2\pi \left(f_{0}+\Delta f\right)\tau_{i}+\varphi \left(\tau_{i}\right)\right]\mathrm{rect}\left[\left(t-\tau_{i}\right)/T_{p}\right] \end{array}$$

The autocorrelation function of I(t) can be written as follows:

(4) $$\begin{array}{cc} R_{\prod }\left(T\right) & =E\left[I\left(t\right)I\left(t-T\right)\right]\\ & ≃2E_{0}^{2}E_{LO}^{2}T_{p}\bar{a^{2}}\exp\left(-2\alpha \nu t\right)\mathrm{tri}\left(T/T_{p}\right)\cos\left(2\pi \Delta ft\right) \end{array}$$

where $\bar{a^{2}}=E\left(a_{i}^{2}\right)$, $\mathrm{tri}\left(T/T_{p}\right)=1-\left|T\right|/T_{p}$ for $\left|T\right|<T_{p}$ and 0 otherwise. According to the Wiener-Khinchin theorem, the instantaneous power spectrum of the beat signal is the Fourier transform of (4) [24], which can be written as:

(5) $$S\left(t,f\right)≃2E_{0}^{2}E_{LO}^{2}T_{p}\bar{a^{2}}\exp\left(-2\alpha \nu t\right){\mathrm{sinc}}^{2}\left[\left(f\pm \Delta f\right)T_{p}\right]$$

where sinc(x)=sin(πx)/(πx). Based on (5), we can obtain the center frequency and the main lobe width of the beat signal, which is given by Δf and $2/T_{p}$ respectively. In addition, the full width at half-maximum (FWHM) of the power spectrum of the beat signal can be estimated to be $0.8859/T_{p}$ according to function ${\mathrm{sinc}}^{2}()$.

### 2.2. Ultra-Low Sampling Resolution and Undersampling Techniques

**Figure 1:**
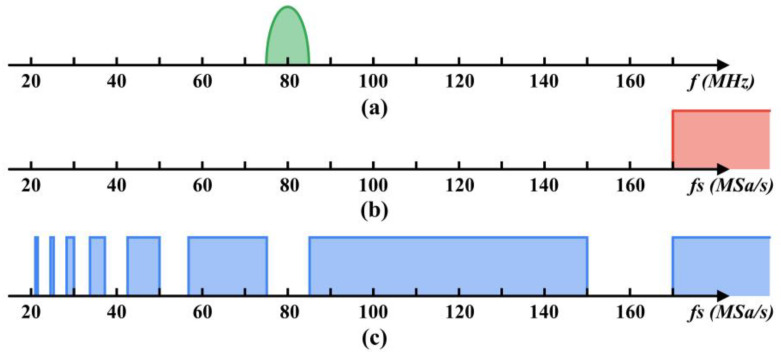
(**a**) A demonstration of the frequency spectrum of the beat signal. The corresponding available sampling rate based on (**b**) the Nyquist–Shannon sampling theorem and (**c**) the undersampling theorem.

Since the beat signal is a bandpass signal whose center frequency is Δf, the undersampling technique can be applied to replace the Nyquist–Shannon sampling theorem during the data-acquisition process in conventional heterodyne Φ-OTDR. The sampling process is a periodic expansion of the signal spectrum at intervals of the sampling rate. By using a selected sampling rate, it is feasible to complete the signal acquisition process while avoiding spectral aliasing at a relatively low sampling rate. According to [24], the range of the undersampling rate $f_{s}$ should satisfy the following inequation:

(6) $$\frac{2f_{U}}{m}\leq f_{s}\leq \frac{2f_{L}}{m-1}$$

where $f_{U}=f_{C}+B/2$ and $f_{L}=f_{C}-B/2$ are the upper and lower cutoff frequencies of the beat signal respectively; $f_{C}$ and B is the center frequency and bandwidth of the beat signal respectively; m is an integer given by $1\leq m\leq f_{U}/B$. As shown in Figure 1, supposing the center frequency and the bandwidth of the beat signal is 80 MHz and 10 MHz respectively, we can obtain the available range of the undersampling rate. Compared to the sampling rate of at least 170 MSa/s required by Nyquist-Shannon sampling theorem, the undersampling theorem provides a wider range of available sampling rates. Specially, a sampling rate lower than the center frequency of the beat signal is also feasible, e.g., 50 MSa/s, which can significantly reduce the number of samples captured by the Φ-OTDR system.

**Figure 2:**
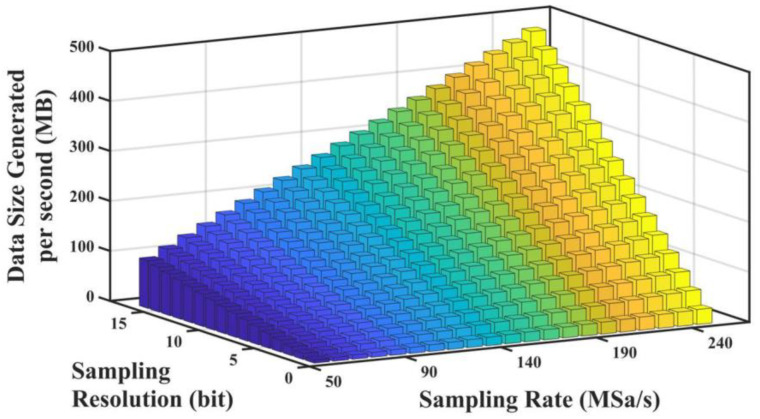
The amount of data generated per second under different sampling rates and sampling resolutions.

As depicted in (3), the impact of external perturbations on the sensing fiber was reflected in the phase variation of the RBS light, and the waveform of the perturbations could be obtained by extracting its phase information. Therefore, the precision of the amplitude of I(t) could be reduced sufficiently to diminish the size of each sample during the data-acquisition process [25]. In summary, the undersampling theorem enabled the use of lower sampling rates for data acquisition, which reduced the number of samples captured per second; on the other hand, the ultra-low sampling resolution technique offered a significant reduction in the file size of individual samples. The amount of data generated per second under the different sampling rates and sampling resolutions is illustrated in Figure 2, and we can see that the sampling rate and sampling resolution had a large impact on the amount of data produced per second. Therefore, combining the ultra-low sampling resolution and undersampling techniques to properly decrease the applied sampling rate and resolution presented a highly promising data-reduction solution for the heterodyne Φ-OTDR system.

**Figure 3:**
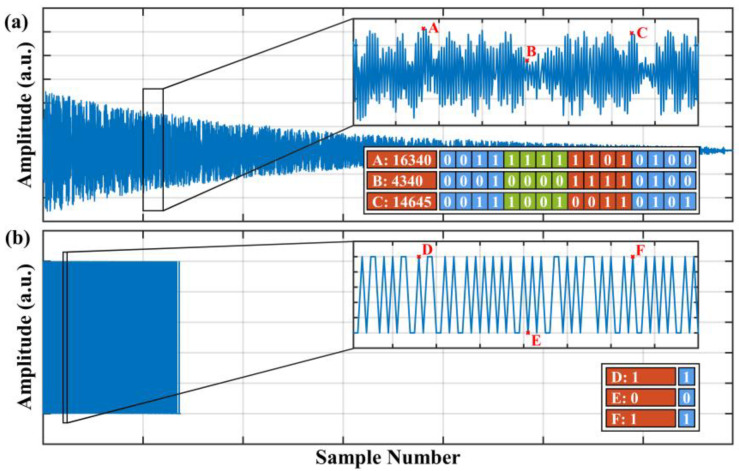
The comparison of (**a**) a conventional RBS trace and (**b**) the corresponding trace after undersampling and 1-bit-resolution processing.

Figure 3 demonstrates the comparison of a conventional trace and the trace after the undersampling and ultra-low-resolution processing. The illustrated conventional trace in Figure 3a was filtered by a bandpass filter, and the sampling rate of this trace was 250 MSa/s. By extracting a fixed-position sample out of every five samples, we obtained the undersampled trace in Figure 3b, whose sampling rate was 50 MSa/s. It could be figured out that the number of samples in the undersampled trace was a fifth of that of the conventional trace. Specifically, the inset in Figure 3a had 400 samples, and the corresponding data in the undersampled trace had only 80 samples, as shown in the inset in Figure 3b. In addition, the samples in Figure 3b were all represented by only 1 bit instead of 16 bits as in Figure 3a, which decreased the storage space by a sixteenth for each sample. The resolution conversion process was implemented by a binary switch, which assigned 1 to samples that exceeded the threshold and 0 otherwise. In addition, the threshold was defined as the average value of this trace. Based on the above discussion, we concluded that the volume of data in the undersampled 1-bit-resolution trace in Figure 3b was an eightieth of that in Figure 3a, which was a considerable reduction rate. It should be noted that this process could be implemented by an inexpensive data-acquisition device with a low sampling rate and a low sampling resolution in practical applications.

## 3. Experimental Setup and Results

### 3.1. Single-Point Sensing Experiment with PZT

**Figure 4:**
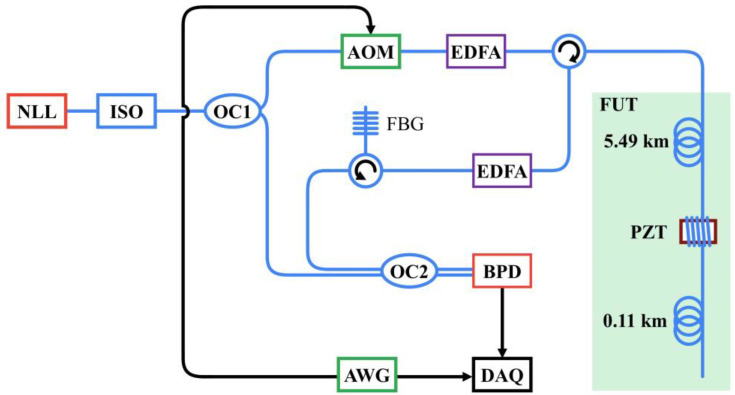
Single-point sensing experimental setup.

A single-point sensing experiment was conducted to verify the feasibility of this data-reduction solution. The experimental setup was a conventional heterodyne Φ-OTDR scheme, as shown in Figure 4. A 5 kHz narrow-linewidth laser (NLL) with 23 mW output power was adopted as the light source, which emitted a continuous light to an isolator (ISO). After passing through the ISO, the continuous light was divided into two parts by a 10:90 optical coupler (OC). Then, 10% of the light acted as the local reference signal and 90% of the light was fed into the acousto-optic modulator (AOM), which turned it into probe pulses with 100 ns pulse widths. The frequency shift of the AOM was 80 MHz, and the repetition rate was set at 16 kHz. The probe pulses were amplified by an Erbium-doped fiber amplifier (EDFA) and then launched into the fiber under test (FUT) through an optical circulator. The FUT was composed of three sections. The first and last parts were single-mode fibers with a length of 5.49 km and 0.11 km, respectively, and the middle section was a homemade piezoelectric transducer (PZT) with a 5 m fiber wrapped around it, which was used to generate the dynamic strain on the FUT. The PZT was driven by a 1 kHz sinusoidal signal with a voltage of 3 V. The RBS light that was returned from the sensing fiber was amplified again by another EDFA and then filtered by an FBG to remove the amplified spontaneous emission noise. The local reference light interfered with the RBS light at a 50:50 coupler, resulting in a beat signal with a center frequency of 80 MHz. The beat signal was then converted to an electrical signal by a balanced photodetector (BPD) with a bandwidth of 300 MHz.

In practical applications, the beat signal is fed into a bandpass filter to remove the undesired frequency components, and then captured by a data-acquisition (DAQ) card with a suitable undersampling rate and a 1-bit resolution. However, to compare the performance of the proposed method and the conventional sampling process with respect to phase demodulation, the same data file needs to be used to ensure the reliability of the experiment. Therefore, applying the conventional sampling process to capture the beat signal and then applying an ultra-low sampling resolution and the undersampling techniques in the digital domain is a reasonable approach. In our experiments, the beat signal output from the BPD was directly captured by a DAQ card, and the sampling rate and resolution were 250 MSa/s and 16 bits, respectively. An arbitrary waveform generator (AWG) was applied to drive the AOM and DAQ card.

**Figure 5:**
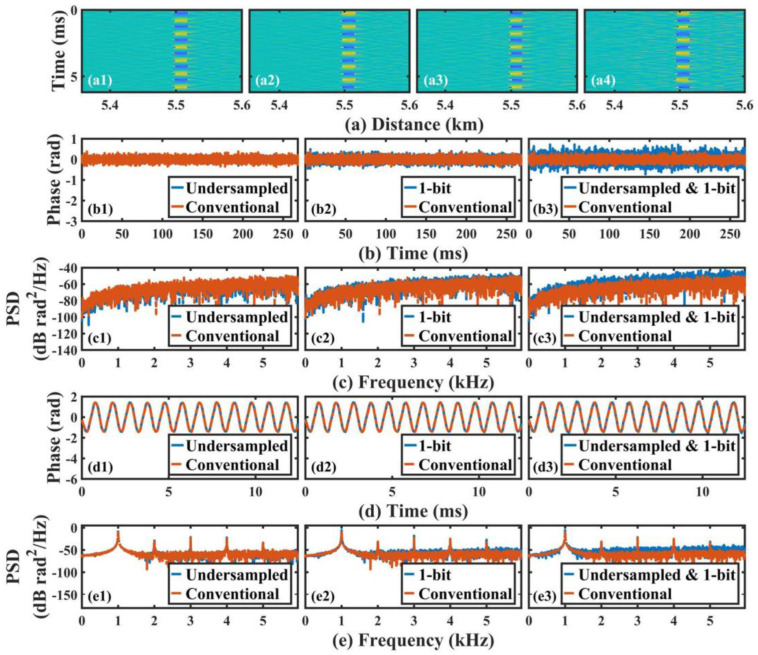
(**a**) Demodulated differential phase by (**a1**) the conventional process, (**a2**) the undersampling technique, (**a3**) the 1-bit sampling resolution technique, and (**a4**) a combination of these two techniques, respectively; (**b**) time-domain phase waveform in the undisturbed fiber section, and (**c**) corresponding PSD results; (**d**) time-domain vibration waveform, and (**e**) corresponding PSD results.

The same data file (128 MB, 268.44 ms sensing time) was utilized to compare the performance of the conventional process, undersampling technique, ultra-low sampling resolution technique, and the combination of these two techniques on phase demodulation; the results are shown in Figure 5. The raw data were first filtered by a bandpass finite impulse response filter with a center frequency of 80 MHz and a 10 MHz passband. Phase demodulation was then carried out. It should be noted that the rotated-vector-sum (RVS) algorithm [28,29,30] was employed to remove the fading noise. This was the conventional demodulation process. Based on this, the undersampling technique was added before the phase demodulation to reduce the sampling rate to 50 MSa/s. Based on the undersampling theorem, the center frequency of the beat signal was shifted to 20 MHz, so the parameters of the filters in the RVS algorithm were adjusted properly according to the undersampling multiplier. In addition, since the number of samples in a trace had changed, the phase differential interval also needed to be modified accordingly to keep the gauge length consistent. Other than that, the rest of the parameters were the same as the conventional process. To carry out the ultra-low sampling resolution technique in the digital domain, the 16-bit raw data were first fed into a binary switch to reduce the resolution to 1 bit. These 1-bit data were then filtered by the same filter as used for the conventional process to restore the waveform, and the subsequent procedures were the same as the conventional process. Regarding the combination of the ultra-low sampling resolution and undersampling techniques, the operation steps are described below. Firstly, the undersampling technique was applied to obtain a 50 MSa/s sampling-rate for the data. The resolutions of these data were then each converted to 1 bit, which resulted in undersampled 1-bit resolution data. It is worth pointing out that the file size was reduced from 128 MB to 1.6 MB, and the space saving ratio reached up to 98.75%. Thirdly, a filter with a 20 MHz center frequency and a 9 MHz passband were applied to restore the waveform of the undersampled 1-bit data. Finally, the phase demodulation was carried out. As mentioned above, the related parameters in the phase demodulation were also modified accordingly compared to the conventional process, and the center frequency of the undersampled 1-bit signal was 20 MHz due to the undersampling operation.

In Figure 5, we can see that all of the data could be used to locate the vibration as well as obtain its waveform, and the demodulated vibration waveform and its frequency were consistent with the driving sinusoidal signal of PZT, which provides a proof of the feasibility of the purposed method. However, from the differential phase results, the time-domain phase waveform at its undisturbed place and its PSD, as well as the vibration PSD, a significant increase could be observed in the noise floor of the undersampled 1-bit data compared to the conventional data.

Specifically, according to Figure 5(b1,b2,b3), the root mean square (RMS) of the phase waveform at the quiet place demodulated from the undersampled 1-bit data was approximately 0.21 rad, while this value was 0.11 rad, 0.08 rad, 0.13 rad for the conventional data, undersampled data, and 1-bit data, respectively. Moreover, the higher noise level of the undersampled 1-bit data was also evident in Figure 5(c3). This result indicated that for phase results in the undisturbed fiber section, the undersampling 1-bit operation would introduce more noise. On the other hand, regarding the vibration waveform, the demodulation results from these four data were not very distinct, as shown in Figure 5(d1,d2,d3). However, the impact of the undersampling 1-bit operation was reflected in the noise floor of the PSD of the vibration signal, as depicted in Figure 5(e3). The vibration SNR (the ratio between the RMS of the noise floor and the peak PSD value) demodulated from the conventional data, undersampled data, 1-bit data, and the undersampled 1-bit data were 65.95 dB, 65.9 dB, 61.8 dB, and 56.13 dB, respectively.

The rise of the noise floor in the low-resolution data might be due to the noise introduced in the resolution conversion process or the misclassification of a valid sample. In addition, the approximate 10 dB reduction in the SNR for the undersampled 1-bit data was likely a result of the decreased number of samples after undersampling, which made the data less robust and, thus, more prone to noise during the resolution conversion process. Considering that this hybrid technique probably deteriorated the system SNR, it might not be suitable for systems where the SNR is not adequate. Optimizing the undersampling and the ultra-low sampling resolution process is a potential way of mitigating this problem. Nevertheless, the vibration localization and waveform analysis were accomplished using this 1.6 MB of data with an acceptable SNR, which efficiently provided a new way to reduce data in the Φ-OTDR system.

### 3.2. Multi-Point Sensing Experiment on a Wooden Board

**Figure 6:**
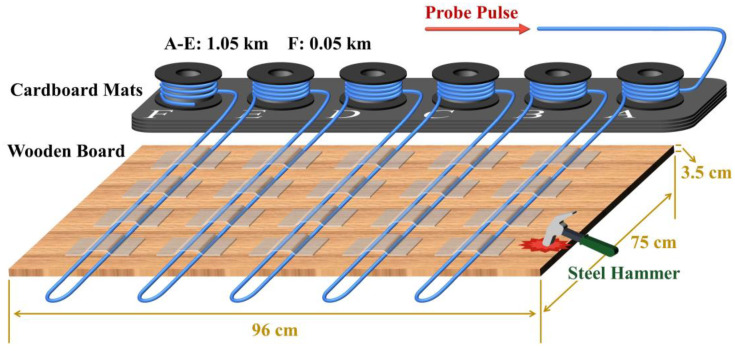
FUT setup in the multi-point sensing experiment.

**Figure 7:**
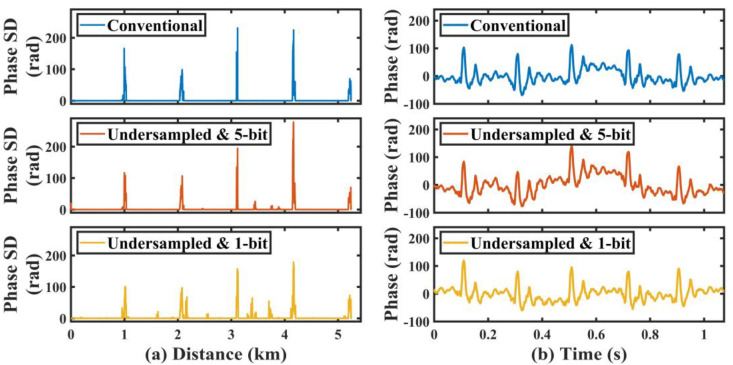
(**a**) Standard deviation (SD) curve of the phase signal (17,179 traces) demodulated from the conventional data, undersampled 5-bit data, and undersampled 1-bit data; (**b**) time-domain vibration waveform at 5.2 km.

To further investigate the effect of the ultra-low sampling resolution and undersampling techniques on system performance, a multi-point sensing experiment was implemented. The experimental setup was basically the same as that of the previous experiment, except for the deployment of the sensing fiber. The total length of the FUT was set at 5.25 km, and it was composed of five sections, each comprised 1 km of standard single-mode fiber and 0.05 km of fiber patch cords. The connection was completed in the order of a 1 km optical fiber followed by a 0.05 km patch cord, and a segment of these optical fiber patch cords were fixed on a wooden board with tape, as illustrated in Figure 6. The length, width, and thickness of this wooden board were 96 cm, 75 cm, and 3.5 cm, respectively. A steel hammer was used to continuously strike the marked area of the wooden board, and the resultant mechanical vibrations were recorded by the Φ-OTDR system. The file size of each acquisition was 512 MB, corresponding to 1.074 s sensing time. The ultra-low sampling resolution and undersampling techniques were applied to these data, and the results are shown in Figure 7.

Figure 7a demonstrates the SD curves of the phase signal that was demodulated from the conventional data, undersampled 5-bit data, and undersampled 1-bit data. In the demodulation results of these three data, we could see that vibrations were detected in all five sections of fiber that were affixed to the wooden board; however, several strong noises appeared in the phase SD curve obtained from the undersampled 1-bit data, which reflected that the combination of the 1-bit sampling resolution and undersampling techniques worsened the SNR of the system. However, when the data resolution was increased to 5 bits, the quality of the phase SD curve was significantly improved. Figure 7b shows the time-domain vibration waveform detected by the fifth section of fiber, and we can see that the vibration pattern of the hammer striking is a kind of damped oscillation. This pattern could be observed in the demodulated vibration waveforms from all three data, which illustrated that vibration waveform analysis could be performed by the undersampled 1-bit data.

## 4. Conclusions

In summary, a data-reduction solution for the Φ-OTDR system based on the combination of the undersampling and the ultra-low sampling resolution techniques was proposed. Two experiments with different FUT deployments were carried out to evaluate the performance of the proposed method on phase demodulation. The results showed that the acoustic signals could successfully be recovered from the undersampled 1-bit data. In addition, the file size of 268.44 ms of sensing data was reduced from 128 MB to 1.6 MB, with a space saving ratio of 98.75%. Although the SNR of the undersampled 1-bit data was not as good as that of the conventional data, this situation could be substantially improved by appropriately increasing the data resolution. With the proposed method, the data acquisition and storage costs of the system were more economical, which could also further promote the commercial applications of Φ-OTDR.

## Data Availability

Not applicable.
